# The role of primary health care services to better meet the needs of Aboriginal Australians transitioning from prison to the community

**DOI:** 10.1186/s12875-015-0303-0

**Published:** 2015-07-22

**Authors:** Jane E. Lloyd, Dea Delaney-Thiele, Penny Abbott, Eileen Baldry, Elizabeth McEntyre, Jennifer Reath, Devon Indig, Juanita Sherwood, Mark F. Harris

**Affiliations:** Centre for Primary Health Care and Equity, Faculty of Medicine, University of New South Wales, Sydney, Australia; Aboriginal Medical Service Western Sydney, Sydney, Australia; Department of General Practice, Faculty of Medicine, University of Western Sydney, Sydney, Australia; School of Social Sciences, Faculty of Arts and Social Sciences, University of New South Wales, Sydney, Australia; Australian Prevention Partnership Centre, University of Sydney, Sydney, Australia; National Centre for Cultural Competence, University of Sydney, Sydney, Australia

**Keywords:** Primary health care, Prisoners, Criminal justice system, Family practice, Aboriginal Australians

## Abstract

**Background:**

Aboriginal Australians are more likely than other Australians to cycle in and out of prison on remand or by serving multiple short sentences—a form of serial incarceration and institutionalisation. This cycle contributes to the over-representation of Aboriginal Australians in prison and higher rates of recidivism. Our research examined how primary health care can better meet the health care and social support needs of Aboriginal Australians transitioning from prison to the community.

**Methods:**

Purposive sampling was used to identify 30 interviewees. Twelve interviews were with Aboriginal people who had been in prison; ten were with family members and eight with community service providers who worked with former inmates. Thematic analysis was conducted on the interviewees’ description of their experience of services provided to prisoners both during incarceration and on transition to the community.

**Results:**

Interviewees believed that effective access to primary health care on release and during transition was positively influenced by providing appropriate healthcare to inmates in custody and by properly planning for their release. Further, interviewees felt that poor communication between health care providers in custody and in the community prior to an inmate’s release, contributed to a lack of comprehensive management of chronic conditions. System level barriers to timely communication between in-custody and community providers included inmates being placed on remand which contributed to uncertainty regarding release dates and therefore difficulties planning for release, cycling in and out of prison on short sentences and being released to freedom without access to support services.

**Conclusions:**

For Aboriginal former inmates and family members, release from prison was a period of significant emotional stress and commonly involved managing complex needs. To support their transition into the community, Aboriginal former inmates would benefit from immediate access to culturally- responsive community -primary health care services. At present, however, pre-release planning is not always available, especially for Aboriginal inmates who are more likely to be on remand or in custody for less than six months.

## Background

Aboriginal Australians are significantly overrepresented in Australia’s criminal justice systems [1]. Whilst Aboriginal people comprise only 2 % of the general Australian adult population, Aboriginal people represented 28 % of the total full-time prisoner population as of March 2014 [2]. Despite federal, state and territory government stated aims to reduce Aboriginal overrepresentation in Australian prisons the rate of Aboriginal incarceration is increasing, especially for Aboriginal women. Between March 2013 and March 2014 the imprisonment rate for Aboriginal males increased by 5 % and for Aboriginal females by 15 % [2].

In addition to the disproportionately high rates of incarceration, Aboriginal Australians are more likely than other Australians to cycle in and out of prison on remand or to serve multiple short sentences [1, 3], which is a form of serial incarceration and institutionalisation [4]. This cycle contributes to a higher rate of recidivism among Aboriginal people when compared to other Australians who offend [5].

In some Aboriginal communities about half the young male population is either in prison or has been in prison [6]. This hyper-incarceration can mean prison moves from being hidden and peripheral to being more common, known, accepted, expected and normalised [7]. Growing up in regular contact with the criminal justice system where many members of the community are or have been incarcerated can result in the distortion of social norms where prison can become a social institution and an anticipated life event [7]. It also means that normal life processes of interacting with siblings, cousins, parents and friends are lost, making it difficult to establish and maintain meaningful relationships with people outside the prison system [8]. This robs people of the opportunity to live a fulfilling life and is another significant factor in the high rates of recidivism among Aboriginal people [5].

A contributing factor to higher recidivism rates is the lack of services available for people on release from prison. Without access to comprehensive health care and social support, Aboriginal people are more likely to return to the same environments that led to their incarceration [5, 9]. Thus social and health determinants such as unemployment, unstable housing, mental illness and drug and alcohol problems remain issues on release [10]. The difficulties former inmates face in managing these issues is compounded by the high emotional stress associated with adjusting to an often chaotic community life.

### Aboriginal inmates’ health needs on transition to the community

In addition to their overrepresentation in the criminal justice system, Aboriginal Australians suffer from significantly poorer health outcomes compared to other Australians [11]. Social factors such as lower education and employment rates contribute to this health gap as do factors such as inadequate access to health services and behaviours such as smoking, poor nutrition and inadequate physical activity [11]. Aboriginal people in prison have higher rates of mental and cognitive disability than non-Aboriginal prisoners [12], and this group experiences higher multiple and complex support needs [13, 14].

To assist prisoners in their transition from custody to community, successful programs require pre-release planning; provision of emotional support from caseworkers, family members and friends; and practical support such as access to stable housing and financial resources. Access to health care is also crucial during this transition, particularly for Aboriginal inmates. Aboriginal people who have been in custody are likely to experience multiple, long standing health issues [15], and to be at a high risk of illness and injury post release [16, 17].

In light of the increased health and other risks for Aboriginal former inmates and significant service gaps, the aim of our research was to develop culturally specific understandings of how primary health care services can better meet the health care and social support needs of Aboriginal Australians transitioning from the criminal justice system into the community. We conducted a qualitative analysis of interviews with eight service providers, 12 former inmates, and ten family members regarding their experiences of services provided to prisoners both during prison and on transition to the community. The purpose of the research was to understand how primary health care can better meet the needs of Aboriginal people released from the criminal justice system, with a view to improving their quality of life and reducing Aboriginal incarceration.

## Methods

The SPRINT acronym stands for Services and Primary health care needs for Recently released Inmates in Need of Treatment and health management. The SPRINT project involved a partnership between Aboriginal and non-Aboriginal researchers. The project was a partnership between the Aboriginal Medical Service Western Sydney (AMSWS) with the University of New South Wales (UNSW), University of Western Sydney (UWS), University of Technology Sydney and the NSW Justice Health and Forensic Mental Health Network. Three of the Chief Investigators were Aboriginal. One of the AMSWS team members was also a researcher at UWS and a Chief Investigator on the project.

The study was conducted in two urban Aboriginal communities in Sydney, New South Wales. The project was funded by the Australian Primary Health Care Research Institute and involved a systematic literature review, analysis of a linked data set assessing hospital use by former inmates and interviews with Aboriginal people released from the criminal justice system, family members and community service providers. This paper reports on the findings from the interviews. However a summary of the literature review findings and methodology is described in the paragraph below.

The systematic literature review was conducted to identify what is known about effective strategies for providing coordinated health care from prison to primary health care in the community for Aboriginal Australians. Electronic databases were the primary source of the literature. A search of Medline, Embase, CINAHL, Criminal Justice Abstracts, Google Scholar and Psych INFO databased identified 1531 papers. After verification against the inclusion criteria 43 studies were included in the review. The review found that the transition from custody to the community can be a time of high vulnerability for Aboriginal former inmates due to a lack of housing and employment, alienation from culture and community, high medical and mental health needs and risk of relapse to substance misuse and risky behaviours. Yet, availability of appropriate pre- and post- programs is patchy and services are poorly coordinated, especially for women, remandees and those who are released to freedom [18].

This qualitative study was led by AMSWS and the UNSW. This was underpinned by a partnership agreement that specified that the data collected by AMSWS remained the property of the community from which it was collected as represented by the Board of Directors of AMSWS. UNSW has an irrevocable, non-transferable license to use the Project Intellectual Property (IP). Both parties committed to the use of IP for the benefit of the Indigenous community. The challenges of this approach included the additional time required to build and maintain a trusting relationship; the ongoing need to manage and reflect upon the expectations among the researchers, the service providers, the funding body and academic institutions. The advantages of this approach are that it sits well within the NHMRC guidelines for ethical research, and increases the likelihood of the successful implementation of recommendations within the community and by service partners because they are invested in the research process. A team of service providers at AMSWS comprising Aboriginal Health Workers, health promotion officers, a general practitioner and the Director of Research were responsible for undertaking the research. Informed consent included an explanation that participation was voluntary and that interviewees were able to withdraw from the research at any time. Former inmates and community members were also paid $40 for their time and to assist with any travel costs associated with the research.

### Ethics

The SPRINT Project was approved by the University of New South Wales Ethics Committee (HC12480), the Aboriginal Health and Medical Research Council’s Ethics Committee (AH&MRC) (874/12) and the Justice Health and Forensic Mental Health Network Human Research Ethics Committee (G388/12). Full informed consent was obtained from all participants.

### Participants

#### How respondents were selected

Purposive sampling was used to identify interviewees who were Aboriginal and had either been in contact with the criminal justice system or had a family member who had been in prison. When selecting former inmates or family members to interview the team of researchers at AMSWS ensured a range of perspectives were sought. Therefore we selected both men and women who were over the age of 18, and people who had been recently released from custody as well as those who had been released some time ago. The team of researchers at AMSWS brainstormed a list of local community service providers who were actively involved in the care of former inmates. Both health care providers and social service providers working for government and non-government organisations were invited to participate.

#### Sample size

Thirty interviews were conducted by the team of health professionals from AMSWS between September 2012 and February 2013. Of these, 12 interviews were with Aboriginal people who had been in prison; ten were with family members and eight with community service providers.

### Procedure

#### What data was collected?

The findings of the systematic literature review were used to inform development of the interview guide. For example the literature review revealed that a great deal more is known about the health and social support needs of Aboriginal people in custody than is known about the needs and experiences of their family, or about inmates’ access to and the effectiveness of post release programs for Aboriginal people. It was therefore considered important to interview family members as well as former inmates and community service providers.

Three separate interview guides were developed by a team of health professionals at AMSWS - one each for Aboriginal former inmates, family members and service providers. The draft interview guides were circulated to chief investigators (CIs) for input and comments.

We focused interview questions on former prisoners’ access to services during the transition from prison to community. Family members were asked what life was like for the family with a relative in prison. They were also asked about their relative’s access to health on release, and the kinds of health services and support that would be most helpful to Aboriginal former inmates and their families at that time. Community service providers were asked about how they work with Aboriginal people leaving custody, factors that assist them in providing effective services and factors that impede them from performing the work that they would like to do.

#### How data was recorded and analysed

Interviews were digitally recorded and transcribed verbatim. All transcripts were read by one researcher to check for accuracy and to remove any identifying information. The transcripts were then reread and notes were handwritten on the right hand side of the transcript. Saturation was reached with no new themes emerging from the final transcripts. Initial codes were drafted and patterns and differences were discussed by two researchers. The initial codes were then collated into tentative themes and the interviews were then reread in order to gather all the relevant data that applied to the tentative themes. A summary description of each of these themes was then drafted and discussed by two researchers with advice and input from another researcher. This enabled us to unpack the themes and identify points of difference. Cross cutting themes over the three groups (former inmates, family members and community service providers) were then identified. This involved developing connections between concepts and categories and to consider these concepts in relation to the existing literature. These common themes were discussed with the team at AMSWS who conducted the interviews to verify accuracy.

## Results

Of the 12 inmates interviewed seven were male and five were female. The majority of prison sentences were for less than 2 years and most participants had a long history, from a young age, of enmeshment in the criminal justice system. The frequency of incarceration varied. Some participants had only been in prison on a couple of occasions, however others had been in prison more than five times.

Of the ten family members interviewed eight were women, and in almost all cases these women were the main, and sometimes the only, support for the former inmates. Where the family member interviewed was a child or partner (rather than an aunt, sister or mother) they seemed to be less aware of the details of the health issues affecting the former inmate on release, and also less able to provide in-depth information about the services that the family member had accessed or had been referred to on release.

Eight service providers were interviewed, four were Aboriginal and all worked for a health or social service community organisation. Four of these organisations were government agencies and the other four were non-government organisations such as charities and community controlled services.

The thematic analysis revealed perceptions among Aboriginal former inmates, family members and community service providers of inadequate in-custody health care and links to the community. Further that access to primary health care on release and during transition was partially dependent on appropriate health care in custody. Different strategies were suggested for those in-custody, during transition and in the community. These are reported below.

### In-custody

Although it was not the main focus of the interview questions, the majority of participants made comments about their health care experience while in custody. Many reported that mental health care in custody tended to focus on medications rather than other interventions. Some family members described feeling relief once their relative was incarcerated because they knew that at least their family member was taking their medication. Others felt that the mental health care in custody was inadequate:

They’re just in there to do their time, and get medicated and keep the calm and out of their hair. More or less, but they don’t get much more help than that. (Family Member—sister)

Community service providers also supported the finding of reliance in custody on mental health medications. Some community service providers commented on the differences between mental health care in custody compared to that provided in the community:

[in the community] we do motivational interviewing to help reframe their life, to give them positive feedback… about their own capabilities. (Mental Health Service Provider)

In addition, CSNSW’s policy of ‘suicide-watch’ was considered to be traumatic and a barrier to seeking appropriate mental health care in custody. One participant reported being on suicide-watch for 2 weeks, after which he was returned to the main prison without being offered any counselling or support. He felt further traumatized by this:

… they put me on a RIT, a suicide-watch. That just stressed things even worse. I was in a cell for 23 and a half hours a day. You only get a half an hour release … Yeah and I had no clothes. I was in my jocks. No smokes. No one to talk to. (Aboriginal man, former inmate)

Another participant recalled when being incarcerated for the first time, she was warned by an experienced inmate not to mention any mental health issues or anxiety because of the danger of being put on suicide-watch. In the view of several participants, there was inadequate continuity of comprehensive health care in the context of complex needs and significant emotional distress and anxiety.

Two other key barriers to accessing health services in-custody, and therefore to effective pre-release and discharge planning, were identified. Short term imprisonment and remand of Aboriginal people means that lives are disrupted, for example by loss of housing and possessions, yet there is insufficient access (if on remand) or time to access the services available in prison. One former inmate was placed in custody on remand for 3 months ‘on trumped up charges’. He explained that being put on remand meant that he lost his house, his social networks, his routine, his access to support services such as counselling and regular Narcotics Anonymous (NA) groups. When placed in custody he reported the following:

I was on remand so I couldn’t do education courses, I couldn’t do anything to prepare myself for the getting out state…. There was no … clarification I was going to get a sentence for how long? And you’re not allowed to progress in the system when you’re on remand. You’ve got—stuck. You’re in quicksand. You can’t do nothing. (Aboriginal man, former inmate)

The former inmate then described feeling devastated when he was released:

I come out to nothing, literally. I had no house, no stuff. I had the clothes that I went into jail with. I was walking about with a garbage bag full of clothes and I had to start from scratch. And that’s what I think disrupted me the most. (Aboriginal man, former inmate)

There was a feeling among participants that the government is concerned with sending people into custody but far less concerned about peoples’ wellbeing or how they might be supported to function in society.

The second major barrier to accessing health services in custody was the paucity of culturally appropriate and targeted health services in custody. For example, some participants commented that in-custody health services were too clinical and did not involve family members. A culturally appropriate health care service would, they said, provide better access to comprehensive and effective health care for Aboriginal Australians in custody.

Interviewees felt that management of mental health issues, including sequelae of exposure to the trauma of incarceration, should be addressed as a high priority prior to release from prison.

A number of service provider felt the suspension of access to Medicare for all prisoners, even if they are on remand, was a breach of human rights (especially for inmates on remand) and was identified as a major barrier for provision of community health in-reach services. The suspension of Medicare also hinders out-reach services to prisoners. For example prisoners cannot attend an Aboriginal Community Controlled Health Service while they are in custody in the same way that they can attend outpatient departments in hospitals.

### Pre-release

Participant responses indicated that discharge planning and communication was variable and hampered by uncertainty regarding release dates and lack of access to Medicare. Communication between prison and community services appeared to depend on whether a person is released to freedom or on parole, or is sentenced or on remand, and also on the duration of imprisonment. Aboriginal former inmates who were in prison for short periods on remand were reported to be far less likely than those exiting from long sentences to experience good linkages between prison and community services. However there was one case where a female former inmate was in prison for 12 months. She was on remand for 10 months and then was sentenced for 12 months, so only had 2 months left to serve. The participant wanted to apply for housing accommodation for her release but you have to apply for transitional housing three months prior to release and at that time she was not aware of her release date.

Service providers and former inmates indicated that uncertainty regarding release dates meant that discharge summaries were not always written and a week’s supply of medication not always provided to inmates on release. This contributes to a lack of continuity of care and places additional pressure on inmates and family members to identify immediate needs and establish links with health and community services.

The majority of service providers indicated that there is a strong need for pre-release planning for all inmates, regardless of the nature of their incarceration (remand or sentenced). The need was identified for connection of inmates with community services prior to their release so that they are better able to access available services and support.

… near the end of that term [of imprisonment], that’s when there should be some real serious work done with that client with regards to setting up the supports ready to go out. So places like Housing should be contacted. The medical centre should be contacted. If they need furniture and stuff, all those things should be ready so that when people get out of jail, they’re not just left and then they’ve got to struggle to re-establish everything again. (Service provider—Housing NSW)

Another service provider suggested that there is a need for coordinated and holistic pre-release planning across all services:

I think what needs to happen, everyone needs to sit down and say, alright, well, this is what’s going to go on [before release]. This is the plan … By a strong team, I’m talking about you have someone from Probation and Parole. You have somebody from the HASI^1^ program … You have somebody from mental health. You have somebody from drug and alcohol. They don’t have to be from the same service, but they have to know what role they’re actually planning. (Service Provider - Aboriginal mental health worker)

### Post-release

Being released from custody was described as a time of high emotional stress for former inmates and their families. Family members felt unsupported while trying to help former inmates adjust to community life and deal with drug use, aggression or mental health issues.

Better access to health care in the community was reported by those who had been in custody on a sentence longer than six months, and by those who felt empowered, usually through family support or a good case worker, to receive the care that they needed. One former inmate reported receiving good support from her parole officer:

‘Oh, they’re good, Probation and Parole. Like she’s been really good to me. She helped me when I went to a refuge and she helped me ring around a few places…. And I’m actually doing an employment pathway plan through Parole, so we do that every Friday and they supply lunch. (Aboriginal women, former inmate)

However, the majority of former inmates were critical about their experiences with being on Parole, reporting a lack of trust between former inmates and parole officers. One former inmate indicated that he did not take parole because he felt as though he would be set up to fail:

‘See sometimes I didn’t take my release date, I done my full term, I didn’t want to get out on parole. Nothing like that. I just done the whole lot. (Aboriginal man, former inmate)

Another former inmate indicated access to quality parole officers is rare:

‘If you find a good one, grab her or grab him with both hands and don’t let go, because they’re rare’. (Aboriginal man, former inmate)

There is a perception that there is a high turn-over of parole officers:

‘My parole officer changed… in the first six months out of prison I had six parole officers’. (Aboriginal man, former inmate)

Regardless of being released to freedom or on parole, the majority of former inmates indicated that housing, transport, employment and social inclusion were all significant issues on release.

….. [Need access to social services] where was can find people to get places and shelter and things that people are going to benefit from… to stay out of jail. (Aboriginal man, former inmate)

Former inmates who had trouble adapting to community life found it difficult to feel safe and to find a place where they felt they belonged. Having stable housing was considered an important part of feeling safe.

One service provider emphasised that in order to be effective, post-release support must be immediate and easily accessible upon release as the immediate post release period is such a chaotic and vulnerable time.

When they first get released make sure you’re in their face. Don’t say come and see me in a week’s time. Actually get there, see the patient, and say, ‘Hey look this is what you need to do.’ Keep them busy for that week…. (Service provider - Aboriginal mental health worker).

The majority of participants reported that there were inadequate links to community services from prison. A young man with mental health problems and who had been in and out of prison for a decade reported never being given a letter or discharge summary or put in contact with a general practitioners or Aboriginal Medical Service on release. Another male former inmate reported seeing a psychiatrist in prison and being on medication. He was given a list of psychiatrists he could see post release, however he only visited one on one occasion but he did not return because attending the appointment made him feel worse. He reported going to see his GPs within a week of being released in order to get further medication for his mental health condition. A female former inmate who had received mental health care in prison reported receiving a week’s supply of medication on release and being referred to the Aboriginal Medical Service and she reported attending that appointment. She also attends counselling once a fortnight at a Drug and Alcohol Healing Centre. The Community Restorative Centre (a non-government agency) referred her to this counselling service, rather than the general practitioner. She finds her own way to the appointment but they drop her home.

The lack of discharge continuity places pressure on service providers who have to make assessments without important information regarding diagnoses made and treatments provided in prison.

Justice Health can help me enormously if I’m making an assessment by providing a good quality discharge summary and the reasoning why a diagnosis has been made in jail. (Community service provider—mental health).

Some service providers commented that whilst good programs are available for Aboriginal people who have been in custody, Aboriginal former inmates are not aware of these services. In addition to the lack of awareness of services, former inmates do not always meet access criteria, which mean that people are turned away.

It is very hard to get into rehab centres. There was one family, almost got them in … And then one of them was still on methadone, and they don’t accept the methadone. … so then that family couldn’t go there. …but they just kind of lose that enthusiasm once they’re put off and told to go somewhere else. (Community service provider—family worker)

Another interview highlighted the importance of services having an ‘open door’ policy:

We need to have people with pathways, even if they’ve [got] stuck for many years in destructive pathways; we need to keep opening doors. I don’t know where I heard the term recently, but ‘no wrong door’, and I think that’s a good—we’ve got to get away from the idea of ‘Oh you don’t belong in our category of people we help, go away’. (Community service provider, mental health)

## Discussion

### Providing appropriate in custody, pre-planning and post-release support

To improve former inmates’ access to primary health care immediately upon release and during their transition to the community, it is critical to provide effective primary health care services to Aboriginal inmates whilst in custody and to properly plan for their release. In-custody there is a need to address health issues such as mental illness, chronic disease, multi morbidities, alcohol and drug issues, and day-to-day illnesses [14, 15]. Pre-release there is a need to build communication between health care providers in custody and in the community. There is also a need to ensure that former inmates access social support services pre-release because access to income and housing impacts on peoples’ social and emotional wellbeing and impacts practically on their ability to attend a primary health care service.

Existing research suggests that the needs of Aboriginal women and men in prison and post-release may be different [9, 10, 19] and that Aboriginal women are more likely to suffer from mental illness than non-Aboriginal women and Aboriginal men [20]. Therefore it is essential to provide customised and tailored support that accounts for these differences.

### Barriers to accessing health services

Some Aboriginal former inmates reported accessing health services in custody and receiving transitional care. This was more likely if they were serving longer sentences and if they had access to supportive case managers post release. However, the participants reported some significant structural barriers to Aboriginal inmates accessing health and other support services both in custody and post-release—including barriers if serving short sentences and being on remand.

This type of incarceration undermines access to effective and appropriate primary health care for Aboriginal people in custody and on release. Our research suggests that being placed on remand appears to cause additional stress and trauma to Aboriginal inmates, exacerbating the already higher rates of mental illness among Aboriginal people on remand. [21]. Heffernan’s research involving a sample of 347 Aboriginal men and 27 Aboriginal women found mental health disorders were more common among the remanded sample (84.4 %) compared to the sentenced sample (70.4 %) [22].

Being placed on suicide watch was of particular concern to a number of interviewees. They described the experience of suicide watch as anxiety-provoking and dehumanizing. This acted as a barrier to accessing health services because inmates were reluctant to reveal feeling depressed or anxious or to acknowledge suicidal thoughts. They described the experience of suicide watch—including being stripped, made to wear just a gown and placed in isolation—as dehumanising–. Some interviewees believed that Corrective Services NSW’s priority was simply to ensure that inmates do not harm themselves, without further consideration of their wellbeing or the need to provide access to mental health or other services. Furthermore the suicide-watch policy was seen to diminish Aboriginal prisoners’ trust in prison health care providers.

In terms of Aboriginal inmates accessing in-custody health services, another significant barrier is the paucity of culturally appropriate health services available to them—particularly with regard to mental health. In addition to medication there is a need for other therapies such as trauma informed care, strengths-based therapeutic counselling, motivational interviewing and goal setting. There is also a need to deal with complex morbidities such as drug and alcohol misuse and chronic disease in a holistic and culturally sensitive way. When there is no time in custody to initiate these therapeutic relationally based services, it is essential to connect former inmates with appropriate services in the community prior to their release.

Aboriginal former inmates need to access more than just primary health care service in custody and post release. Aboriginal inmates need access to social and family supports as well as access to health care services. Social and family supports can be divided into different types: emotional, informational and instrumental [23]. Emotional support involves listening and providing empathy. Informational supports include receiving advice about the services available. Instrumental support includes the provision of services or goods such as finances, housing or furniture. (Primary health care may have a role in facilitating access to instrumental support such as housing).

This research highlights that all of these supports are necessary to assist people to access services in the community. A well-integrated and coordinated approach to supporting Aboriginal inmates in custody and upon release is therefore essential.

### Lack of continuity and absence of coordinating agency

We found that there is inadequate continuity of comprehensive health care for Aboriginal inmates who suffer significant emotional distress and anxiety and who often have complex health and social needs. Notably, in spite of their particular vulnerability, there is no single agency with responsibility for Aboriginal former inmates’ transition to community. Rather a patchwork of government and non-government services provide piecemeal services to some former inmates, with their family and friends expected to provide all other supports. This finding is supported by results from a 2005 survey undertaken by the Attorney General’s Department, which compiled an inventory of services made available to prisoners in Australia. They found that some—but not all—programs involved multiple service providers from government and non-government organisations, and that not all agencies communicated with each other. The authors therefore argued for increased interagency coordination [24].

Our findings regarding the inadequate continuity of care for Aboriginal inmates in custody and post release are also supported by the 2004 Social Justice Report, which focused on the needs of Aboriginal women in prison. The report found a lack of continuity of care for Aboriginal women between prison and the community, and between support organisations with responsibility for assisting Aboriginal women in the community. It recommended that each state and territory designate a coordinating agency to provide a whole-of-government approach to meeting the needs of Indigenous women leaving prison, and suggested the Department of Justice or the Attorney-General’s Department would be well placed to take on this role [19].

This lack of continuity of care means that many Aboriginal inmates and former inmates are not aware of the services available to them on release. Many Aboriginal inmates with chronic and complex multi morbidities are released without a discharge summary or plan, or without being provided with a connection to community primary health care services.

Aboriginal Australians transitioning from the criminal justice system to the community are therefore a particularly vulnerable group who face multiple financial and social barriers to accessing primary health care. They are often stigmatised and socially excluded [10]. There are poor linkages between health services in custody and culturally-appropriate primary health care in the community. Aboriginal inmates also generally lack trust in government services [25].

Despite the principle of universal access, many Aboriginal people are excluded from accessing many mainstream services in Australia [26]. Aboriginal community controlled health services (ACCHSs) were established to address some of the barriers to effective service provision through culturally appropriate care. ‘Aboriginal communities operate over 150 ACCHSs in urban, regional and remote Australia. They range from large multi-functional services employing several medical practitioners and providing a wide range of services, to small services which rely on Aboriginal Health Workers and/or nurses to provide the bulk of primary care services, often with a preventive, health education focus’ [27]. But Aboriginal community controlled services are not available to all communities or people, although where they have been established; they constitute a significant component of the health care system.

Immediate practical barriers to Aboriginal prisoners accessing primary health care services on release include managing competing priorities such as meeting parole requirements; finding accommodation; reconnecting with family; and securing employment. Former inmates do not always have access to a Medicare Card, Close the Gap measures, or health checks on release, and might not have money to get prescriptions filled. The Close the Gap strategy was developed in 2007 after the Council of Australian Governments signed a statement of intent to meet health targets that aim to close the gap in life expectancy between Aboriginal and non-Aboriginal Australians. Reduced co-payments on prescriptions for Aboriginal Australians with chronic disease are one of the interventions under this strategy.

Prisoners in Australia are suspended from the public health insurance system—Medicare—whilst in prison. They receive health services from the specialist justice health service, in NSW this is called Justice Health &Forensic Mental Health Network (JH&FMHN) which is funded by the state rather than the federal government [28].

The roles of Corrective Services NSW (CSNSW) and JH&FMHN in preparing Aboriginal inmates for release are distinct. CSNSW is responsible for managing the safety and social welfare of people pre-custody, while held in police cells and in-custody; and for providing programs to inmates to assist in addressing offending behaviours. The overarching aim of CSNSW is ‘to reduce reoffending and enhance community safety’.^2^ Corrective Services also has the responsibility of supporting the reintegration of former inmates into the community by providing access to appropriate services and support upon their release. However CSNSW is not currently providing this service to all former inmates—rather it is largely focused on those released on parole.

One of JH&FMHN’s goals is to facilitate continuity of health care for offenders and ex-prisoners on orders in the community.^3^ JH&FMHN aims to facilitate continuity of health care for offenders by providing discharge summaries. However this is not always achieved in the context of remand and uncertainty regarding release dates.

When we presented our findings to the Aboriginal Medical Service at the Community Workshop, some of the participants commented that the trauma caused by incarceration can be compounded by not being able to attend family funerals or celebrate important cultural events. This suggests that providing culturally appropriate health services and access for all Aboriginal inmates to cultural events and cultural connections while in custody are relevant to improving Aboriginal inmates’ health and wellbeing.

There is an opportunity for community primary health care services to work more closely with CSNSW and the JH&FMHN to support Aboriginal inmates’ and former inmates’ access to culturally responsible and comprehensive health care. Community primary health care services are capable of providing in-reach services to Aboriginal inmates in custody, to contributing to release planning and are able to be immediately available to former inmates on their release. Realising this opportunity may require strong advocacy from primary health care professionals and organisations, as well as structural changes including to the use of Medicare. Without these changes the primary health care needs of Aboriginal inmates and former inmates will suffer. This will exacerbate existing poor health and contribute to ongoing higher rates of recidivism and incarceration among Aboriginal Australians.

One of the great strengths of this research was that it was conducted in partnership with an Aboriginal Community Controlled Health Organisation and further that a number of the service providers from the organisation were directly involved in designing and conducting the research. This meant that the interviewers often knew the interviewees well. However the disadvantage of this approach was that interviewees may have been aware of the interviewer in their care provider role and felt less able to criticise the health service or providers in their responses. The interviews also relied on self-report.

## Conclusions

For Aboriginal former inmates and family members, release from prison was a period of significant emotional stress and commonly involved managing complex needs. At present pre-release planning and post release support is not always available, especially for Aboriginal inmates who are more likely to be on remand or in custody for less than 6 months. The findings indicate that Aboriginal former inmates would benefit from immediate access to culturally responsive community primary health care services to support their transition into the community.

Access to comprehensive post release support is dependent upon comprehensive services being provided in custody, pre-release and post release and being able to deal with complex multi-morbidities such as mental illness, cognitive impairment, drug and alcohol misuse and chronic disease. This is a significant undertaking that would rely upon commitment and input from many agencies, including primary health care, and the identification and support of a coordinating agency with responsibility for Aboriginal former inmates’ transition to community. These system changes are required if primary health care services are to help reduce the overrepresentation of Aboriginal Australians in prison.

## Footnotes

1 The Housing and Accommodation Support Initiative (HASI) is a partnership between NSW Health, Housing NSW and various non-government organisations. The initiative provides people with mental health problems access to stable housing linked to clinical and psychosocial rehabilitation services.

2 Corrective Services NSW website; www.corectiveservices.justice.nsw.gov.au.

3 Justice Health & Forensic Mental Health Network website. www.justicehealth.nsw.gov.au.
